# Portal Hypertensive Gastrointestinal Disease in Liver Cirrhosis: The Diagnostic Value of Contrast-Enhanced Multi-Slice Spiral CT

**DOI:** 10.2174/0115734056486778260820080937

**Published:** 2026-08-31

**Authors:** Ning Li, Hui Liu, Xing Lan, Yongkang Liu, Jing Zhou, Jianhua Wang, Zhongqiu Wang

**Affiliations:** 1 Department of Radiology, Affiliated Hospital of Nanjing University of Chinese Medicine (Jiangsu Province Hospital of Chinese Medicine), Nanjing, China; 2 Department of Ultrasonic Medicine, Affiliated Hospital of Nanjing University of Chinese Medicine (Jiangsu Province Hospital of Chinese Medicine), Nanjing, China

**Keywords:** Liver Cirrhosis, Multi-Slice Spiral CT, MSCT, Portal hypertension, Portal hypertension gastropathy, Portal hypertension enteropathy

## Abstract

**Objective:**

This study was designed to explore the diagnostic value of contrast-enhanced multi-slice spiral computed tomography (MSCT) in portal hypertensive gastrointestinal disease (PHGD) among patients with liver cirrhosis.

**Methods:**

A retrospective analysis was performed on cirrhotic patients admitted to our hospital from January 2020 to December 2024. Using endoscopic findings as the reference standard, these patients were divided into the PHGD group and the control group (non-PHGD group). Imaging parameters and serum markers were measured, and differences between the two groups were compared.

**Results:**

A total of 69 PHGD patients and 71 non-PHGD patients were enrolled. In the PHGD group, platelet count (PLT), hemoglobin (HB), and total protein (TP) were significantly lower than those in the non-PHGD group (*P* < 0.05). Regarding imaging indicators, portal vein diameter (PVD), superior mesenteric vein diameter (SMVD), splenic vein diameter (SVD), stomach wall thickness (SD), and stomach wall enhancement thickness (ST) in the PHGD group were significantly greater than those in the non-PHGD group (*P* < 0.05). The diagnostic efficacy of individual imaging parameters was moderate to good, with AUCs ranging from 0.742 to 0.880.

**Discussion:**

Contrast-enhanced MSCT parameters, particularly SMVD (AUC 0.880), PVD (AUC 0.852), and ST (AUC 0.849), demonstrated good diagnostic performance for identifying PHGD in cirrhotic patients. Imaging findings such as venous dilation, splenomegaly, gastrointestinal wall thickening, and ascites were significantly associated with PHGD, suggesting that MSCT may serve as a valuable non-invasive adjunctive tool when endoscopy is unavailable or inconclusive.

**Conclusion:**

PHGD is a common and significant complication of portal hypertension, but there is a lack of standardized clinical diagnostic criteria. Contrast-enhanced MSCT shows considerable potential in assisting the diagnosis of PHGD.

## 1 INTRODUCTION

Cirrhosis is a chronic liver disease triggered by one or more etiological factors, characterized by hepatocyte necrosis, nodular regeneration of residual hepatocytes, formation of fibrous septa, and the development of pseudolobules [1]. A key pathological consequence of cirrhosis is the elevation of portal blood flow resistance, which further leads to portal hypertension (PH). Currently, the hepatic venous pressure gradient (HVPG) is recognized as the gold standard for diagnosing PH. In patients with chronic liver disease, an HVPG >5 mmHg (1 mmHg = 0.133 kPa) is generally considered indicative of PH, while an HVPG ≥10 mmHg signifies clinically significant portal hypertension (CSPH) [2].

Portal hypertension can affect the entire gastrointestinal tract through multiple pathways. On one hand, it alters gastrointestinal hemodynamics; on the other hand, it creates a complex biochemical microenvironment characterized by elevated levels of reactive oxygen species, nitric oxide, tumor necrosis factor-α (TNF-α), endothelin-1, prostaglandins, and other inflammatory mediators. These factors collectively induce cellular damage, leading to mucosal injury in the digestive tract. Such pathological processes trigger a series of mucosal changes and histological abnormalities, ultimately resulting in portal hypertensive gastrointestinal disease (PHGD) [3]. PHGD is a spectrum of diseases that includes portal hypertensive gastritis (PHG), portal hypertensive enteropathy (PHE), and portal hypertensive colopathy (PHC) [3, 4].

Endoscopy is currently the gold standard for diagnosing PHGD. However, its clinical application is limited by several factors: first, the endoscopic manifestations of PHGD are diverse and lack specificity, leading to the absence of unified diagnostic criteria; second, in cases of severe esophageal varices or due to constraints in patient condition or local medical resources, techniques such as capsule endoscopy or small intestinal endoscopy may be unavailable, which significantly restricts the diagnostic rate [4, 5]. Therefore, there is an urgent need to explore non-invasive diagnostic methods that are readily accessible.

Against this background, this study aims to investigate whether dynamic contrast-enhanced multi-slice spiral computed tomography (MSCT) can provide valuable diagnostic information for portal hypertensive gastropathy secondary to cirrhosis, thereby offering a potential non-invasive alternative for clinical practice.

Previous studies have investigated CT findings in portal hypertension, including variceal detection and splanchnic vessel measurements. However, few studies have systematically evaluated the CT characteristics of PHGD as a spectrum involving the entire gastrointestinal tract. Cicero G *et al*. described CT and MR enterography findings in portal hypertensive enteropathy, focusing on small bowel changes in a limited cohort [6]. To our knowledge, no large-scale study has systematically evaluated contrast-enhanced MSCT findings across the stomach, small bowel, colon, and rectum in PHGD patients.

## 2 MATERIALS AND METHODS

This retrospective study adhered to the ethical guidelines of the 1975 Declaration of Helsinki and was approved by the ethics committee of Affiliated Hospital of Nanjing University of Chinese Medicine (Jiangsu Province Hospital of Chinese Medicine), Nanjing, China (Approval No.: 2024NL-KS052). Due to its retrospective nature, the requirement for informed consent was waived.

### 2.1 Study Sample

A retrospective analysis was conducted on patients with liver cirrhosis admitted to our hospital from January 2020 to December 2024. Using endoscopic findings as the reference standard, the patients were divided into the PHGD group and the non-PHGD control group. The inclusion criteria were as follows: (1) The diagnosis of cirrhosis was confirmed in accordance with the *2020 Evidence-Based Clinical Practice Guidelines for Cirrhosis* [7]; (2) All patients had complete serological and abdominal contrast-enhanced CT imaging data. The exclusion criteria were: (1) A history of liver surgery, splenectomy, or gastrointestinal surgery; (2) A history of inflammatory bowel disease; (3) A history of heart failure or renal failure; (4) A history of abdominal malignant tumors; (5) A history of contrast agent allergy.

#### 2.1.1 Endoscopic Diagnostic Criteria for PHGD

PHGD was diagnosed based on established endoscopic criteria: (1) Portal hypertensive gastropathy (PHG) was defined according to the New Italian Endoscopic Club (NIEC) criteria, including mosaic-like pattern (mild) and red spots or black-brown spots with or without mosaic pattern (severe); (2) Portal hypertensive enteropathy (PHE) was defined as the presence of vascular lesions (ectasia, telangiectasia, red spots, or varices) or non-vascular lesions (edema, erythema, erosion, or villous atrophy) in the small intestine; (3) Portal hypertensive colopathy (PHC) was defined as vascular lesions (ectasia, telangiectasia, red spots, or varices) or non-vascular lesions (edema, erythema, erosion, or ulceration) in the colon or rectum, in the absence of other identifiable causes.

All patients underwent upper gastrointestinal endoscopy. Patients with clinical suspicion of lower gastrointestinal involvement (hematochezia, melena, or unexplained anemia with negative upper endoscopy) underwent colonoscopy. Small bowel evaluation was not performed routinely; however, in patients with persistent obscure gastrointestinal bleeding, capsule endoscopy was performed when clinically indicated. For the purposes of this study, the reference standard for PHGD diagnosis was endoscopy (upper and/or lower). Patients with positive endoscopic findings for PHG, PHE, or PHC were classified as the PHGD group. Patients with cirrhosis but no endoscopic evidence of PHGD (normal mucosa or lesions attributable to other causes) were classified as the non-PHGD control group [8, 9].

### 2.2 CT Scanning Protocol

CT examinations were performed using GE Lightspeed 64 VCT and Philips Brilliance 64 iCT scanners. All patients underwent both plain and three-phase enhanced CT scanning. The scanning parameters were set as follows: tube voltage of 120 kV, tube current of 200-400 mA, slice thickness and reconstructed slice interval of 1.0 mm, pitch of 0.984-1.375, and rotation speed of 0.5 s/rotation. For enhanced scanning, 100-120 ml of non-ionic contrast agent iodixanol (350 mgI/ml) was used, and images were acquired in the arterial phase (30 s), portal phase (70 s), and delayed phase (120 s). The scanning range covered from the apex of the diaphragm to the inferior margin of the pubic symphysis.

### 2.3 Image Analysis

Two experienced radiologists, with intermediate and senior professional titles, specializing in abdominal imaging, independently measured the relevant data without access to the patients' clinical information. Prior to the measurements, they received standardized training on the assessment methods, including measuring the portal vein diameter (PVD), superior mesenteric vein diameter (SMVD), and splenic vein diameter (SVD) at the hepatic hilum level and before the confluence of the portal vein, measuring the splenic diameter (SD) and splenic thickness (ST) at the level where the spleen was the largest in size. In addition, they evaluated the presence of portal vein thrombosis, the location and severity of varicose veins, and recorded the changes in the wall structure and adjacent mesentery of the stomach and intestines in patients with PHGD. In case of any discrepancies, a consensus was reached through joint discussion. Furthermore, Cohen’s kappa was used to evaluate consistency between the two, yielding a value of 0.828.

#### 2.3.1 CT Criteria for Jejunal Involvement

Jejunal involvement on CT was defined based on the presence of at least two of the following criteria: (a) small bowel wall thickening >3 mm, (b) mural stratification or ‘target sign’ on contrast-enhanced images (indicating submucosal edema), (c) luminal dilatation with fluid accumulation, (d) engorgement of vasa recta (vascular prominence), and (e) mesenteric edema or haziness. These criteria were applied in consensus by the two radiologists.

Representative imaging and endoscopic findings are illustrated in Figs. (**1**-**4**). Figure **1** demonstrates endoscopic features of PHG (mosaic pattern with hemorrhagic spots). Figure **2** shows colonoscopic findings in PHC (mucosal erythema and vascular ectasia). Figures **3** and **4** illustrate the characteristic CT findings of PHGD, including small bowel wall thickening with the ‘target sign’ (arrowhead), mesenteric haziness, ascites (star), varices (solid arrow), gastric wall edema (dashed arrow), and concurrent splenomegaly with infarction (curved arrow). These representative images were obtained from a 51-year-old female patient with endoscopically confirmed PHG from our cohort.

### 2.4 Statistical Analysis

SPSS 20.0 software was used for data analysis. Quantitative data were tested for normality. Normally distributed data were expressed as mean ± standard deviation. The independent-samples t-test was used to compare the two groups for normally distributed quantitative data. For categorical data, the chi-square (χ^2^) test for contingency tables or Fisher's exact test was conducted. Diagnostic efficacy parameters, including sensitivity and specificity, were computed, and the corresponding receiver operating characteristic (ROC) curves were plotted. A *p*-value < 0.05 was considered statistically significant.

Due to the relatively small sample size (n=140) and the risk of overfitting, a multivariable logistic regression model was not constructed. Therefore, we report the diagnostic performance of individual parameters, which is more robust given the sample size.

## 3 RESULTS

### 3.1 Study Sample

A total of 69 patients were enrolled in the PHGD group, including 34 males and 35 females, with a mean age of 66.6±10.4 years. The non-PHGD group consisted of 71 patients, including 36 males and 35 females, with a mean age of 69.3±9.6 years (Table **1**).

### 3.2 Differences in Laboratory Test Results Between the Two Groups

Laboratory test results showed that platelet count (PLT), hemoglobin (HB), and total protein (TP) in the PHGD group were significantly lower than those in the non-PHGD group (all *p* < 0.05) (Table **2**). Aspartate transaminase (AST) levels in the PHGD group were higher than those in the non-PHGD group (t=2.291, *p* = 0.025), while no significant difference was observed in alanine transaminase (ALT) levels between the two groups (t=0.947, *p* = 0.346). The diagnostic efficacy of PLT, HB, TP, and AST is summarized in Table **3**. Among patients who underwent Stool Routine Tests (SRT), 51 cases in the PHGD group and 54 cases in the non-PHGD group were included. Occult blood was positive in 17 cases (33.3%) and negative in 34 cases (66.7%) in the PHGD group; in the non-PHGD group, occult blood was positive in 16 cases (29.6%) and negative in 38 cases (70.4%). There was no significant difference in the positive rate of occult blood between the two groups (χ^2^=0.167, *p* = 0.683).

### 3.3 Differences in Imaging Findings Between the Two Groups

Imaging measurements revealed that portal vein diameter (PVD), superior mesenteric vein diameter (SMVD), splenic vein diameter (SVD), splenic diameter (SD), and splenic thickness (ST) in the PHGD group were significantly greater than those in the non-PHGD group (all *p* < 0.05). In addition, the PHGD group had a significantly higher incidence of varicose veins, ascites, and portal system thrombosis compared with the non-PHGD group (all *p* < 0.05) (Table **4**). The diagnostic efficacy of PVD, SMVD, SVD, SD, and ST is detailed in Table **3**.

### 3.4 CT-detected Lesion Distribution

Based on CT imaging criteria, CT-detected abnormalities suggestive of portal hypertensive enteropathy were observed in multiple segments: jejunal changes were noted in all 69 cases (100% of the PHGD group), followed by the stomach (41 cases, 59.4%), duodenum (39 cases, 56.5%), ileum (33 cases, 47.8%), colon (24 cases, 34.8%), and rectum (19 cases, 27.5%). It is important to emphasize that these findings reflect CT-detected abnormalities suggestive of portal hypertensive enteropathy, not endoscopic confirmation. Conventional CT has limitations in detecting mild or early mucosal changes, and jejunal involvement was not systematically confirmed by capsule endoscopy [10]. ROC curve analyses for individual parameters were performed using Youden’s index to determine optimal cut-off values. The corresponding ROC curves are shown in (Figs. **5** and **6**).

## 4 DISCUSSION

Portal hypertension (PH) is the initiating factor for portal hypertensive gastrointestinal disease (PHGD) [11,12]. Elevated portal pressure increases intestinal blood flow, leading to dilation and increased permeability of mucosal and submucosal capillaries, resulting in plasma exudation, extensive edema, congestion, and erosion [13,14]. Mucosal ischemia, hypoxia, and metabolic disturbances disrupt intestinal epithelial function, compromise the mucosal barrier, and promote bacterial translocation and inflammation, collectively driving PHGD pathogenesis [15-17].

Endoscopic findings in PHGD are primarily categorized into vascular and non-vascular lesions [18-20]. Vascular lesions include vascular malformations, dilatation, and varices, while non-vascular lesions manifest as inflammation, erythema, velvet-like ectasia, salmon-egg-like changes, and polypoid lesions (Figs. **1** and **2**). Corresponding imaging features include widespread edema and thickening of the gastrointestinal mucosal and submucosal layers (characterized by a “target sign” on contrast enhancement), mesenteric effusion and blurring, as well as varices and ascites (Figs. **3** and **4**).

In this study, platelet count (PLT), hemoglobin (HB), and total protein (TP) were significantly lower in the PHGD group (*p* < 0.05), with moderate diagnostic efficacy (AUCs: 0.797, 0.805, 0.685). These findings reflect advanced cirrhosis with hypersplenism, bleeding, and malabsorption [21-24]. Thrombocytopenia in PHGD is driven by splenomegaly and reduced thrombopoietin production [25-27]. We acknowledge that these laboratory differences are expected in advanced cirrhosis and portal hypertension, and are not specific markers of PHGD per se; their limited individual AUCs (0.642-0.805) support this distinction.

AST was higher in the PHGD group (*p* = 0.025) but had limited diagnostic value (AUC 0.642). ALT showed no significant difference. No significant difference in stool occult blood was observed between groups (χ^2^=0.167, *p* = 0.683).

Imaging parameters, PVD, SMVD, and SVD, were significantly greater in PHGD (*p* < 0.05), with superior diagnostic efficacy (AUCs: 0.852, 0.880, 0.825), indirectly indicating higher portal pressure. The PHGD group also had significantly higher incidences of varices (89.86%, χ^2^=66.381), ascites (χ^2^=71.499), and portal system thrombosis (χ^2^=21.254), consistent with prior reports [28-30]. Portal hypertensive enteropathy (PHE) is thought to develop not solely as a direct consequence of elevated portal pressure, but rather when congestion-induced mucosal injury exceeds an individual susceptibility threshold. This threshold is modulated by the adequacy of collateral venous drainage [31]. Once this threshold is surpassed, a cascade of congestive damage may ensue, aggravating gastric lesions and extending to the small intestine. This concept helps explain the heterogeneous distribution of PHGD lesions observed in our cohort.

Splenic diameter (SD) and splenic thickness (ST) were significantly larger in PHGD (*p* < 0.05; AUCs 0.742 and 0.849), supporting their role as surrogate markers of PHGD severity, consistent with CT volumetry studies [32].

In this cohort, based on CT imaging criteria, CT-detected abnormalities suggestive of portal hypertensive enteropathy were observed in multiple segments: jejunal changes in all 69 cases (100%), followed by stomach (59.4%), duodenum (56.5%), ileum (47.8%), colon (34.8%), and rectum (27.5%). This distribution may relate to regional differences in portal perfusion and venous drainage. It is important to emphasize that these findings reflect CT-detected abnormalities, not endoscopic confirmation; conventional CT has limited sensitivity for mild mucosal changes [10].

Our findings are consistent with those of Nishino K *et al*. [28] (high prevalence of esophagogastric varices in PHG) and Heo *et al*. [33] (PVD measurements in CSPH). Our study extends previous work by systematically evaluating multi-segmental gastrointestinal involvement and quantifying diagnostic cut-off values for multiple imaging parameters.

The proposed cut-off values (*e.g*., PVD >14.35 mm, SMVD >11.75 mm, ST >47.35 mm) demonstrate moderate to good diagnostic performance but have limitations, including single-center derivation, and they cannot replace endoscopy for direct mucosal visualization. Endoscopy remains the gold standard for early or mild PHGD changes.

The diagnostic efficacy of individual imaging parameters was moderate to good, with SMVD showing the highest AUC (0.880), followed by PVD (0.852) and ST (0.849). These results suggest that contrast-enhanced MSCT parameters can assist in identifying cirrhotic patients with PHGD.

We did not construct a combined diagnostic model due to the limited sample size (n=140) and risk of overfitting. Therefore, reported diagnostic performance is limited to individual parameters. The high prevalence of CT-detected jejunal abnormalities (100%) should be interpreted with caution; dedicated CT enterography or MR enterography may provide superior small bowel evaluation.

## 5 STUDY LIMITATIONS

This study has the following limitations: (1) The retrospective design may have introduced selection bias; (2) The relatively small sample size (140 cases) may have limited the generalizability of the findings; (3) Potential measurement errors may have occurred despite standardized training; (4) The reported 100% prevalence of CT-detected jejunal abnormalities in the PHGD group was based solely on CT imaging criteria without systematic endoscopic correlation (*e.g*., capsule endoscopy). Conventional contrast-enhanced CT has limited sensitivity for subtle mucosal lesions, and this finding likely represents overestimation. Future studies should incorporate small bowel endoscopy to validate CT findings; (5) Not all patients underwent complete gastrointestinal endoscopic evaluation. Isolated small bowel PHE without upper or lower tract involvement may have been missed in patients who did not undergo capsule endoscopy, potentially biasing results toward the null. However, this would not explain the positive findings we observed.

## CONCLUSION

In summary, PHGD is a critical complication of portal hypertension, with no standardized clinical diagnostic criteria currently available. Contrast-enhanced MSCT shows promising diagnostic value; cirrhotic patients with abnormal laboratory test results accompanied by dilation of the portal vein, superior mesenteric vein, or splenic vein, splenomegaly, gastrointestinal edema, varicose veins, or ascites should raise strong suspicion for PHGD.

## Figures and Tables

**Fig. (1):**
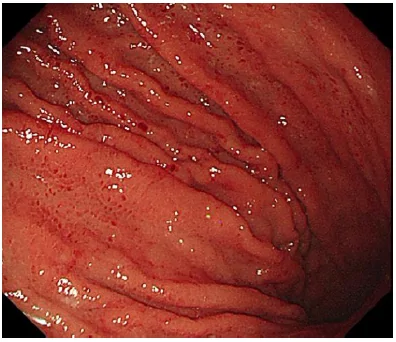
Endoscopic image of portal hypertensive gastropathy (PHG) in a 51-year-old female patient with liver cirrhosis. The image demonstrates gastric mucosal hemorrhage, erythema, and characteristic “salmon egg-like” changes.

**Fig. (2):**
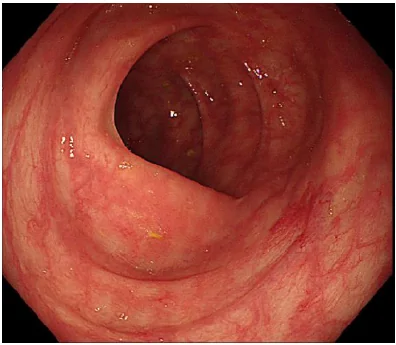
Colonoscopic image of portal hypertensive colopathy (PHC) in the same patient. The image reveals colonic mucosal hemorrhage and erythematous lesions consistent with portal hypertensive colopathy.

**Fig. (3):**
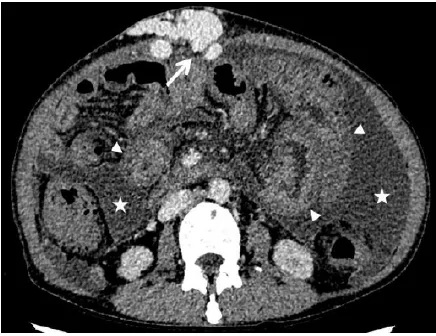
Axial contrast-enhanced CT scan of the same patient showing extensive edematous changes in the small intestine (arrowhead), mesenteric effusion, and significant ascites (star). Prominent abdominal wall varices are also observed (solid arrow).

**Fig. (4):**
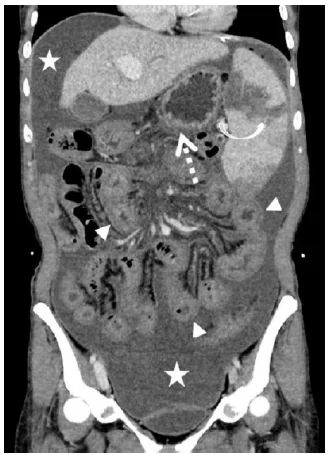
Coronal contrast-enhanced CT scan of the same patient demonstrating gastric wall thickening with edema (dashed arrow), extensive small intestinal edema (arrowhead), mesenteric effusion, and significant ascites (star). Concurrent splenomegaly and splenic infarction are noted (curved arrow).

**Fig. (5):**
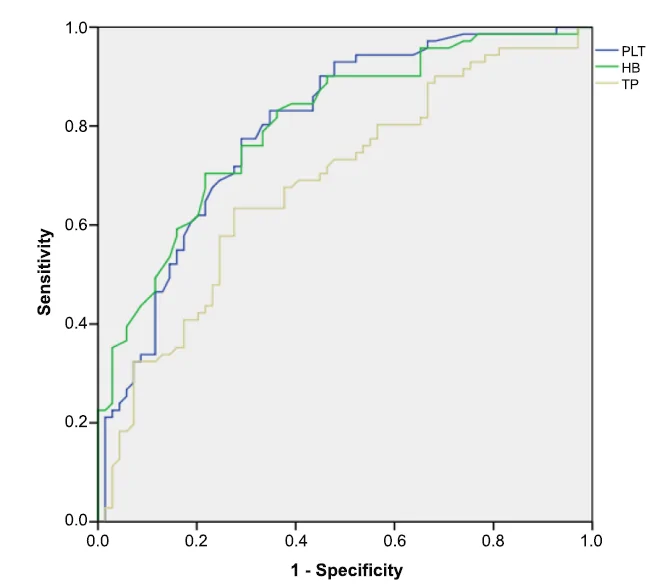
Receiver operating characteristic (ROC) curves for laboratory parameters (PLT, HB, TP, and AST) in predicting PHGD. AUCs: PLT 0.797, HB 0.805, TP 0.685.

**Fig. (6):**
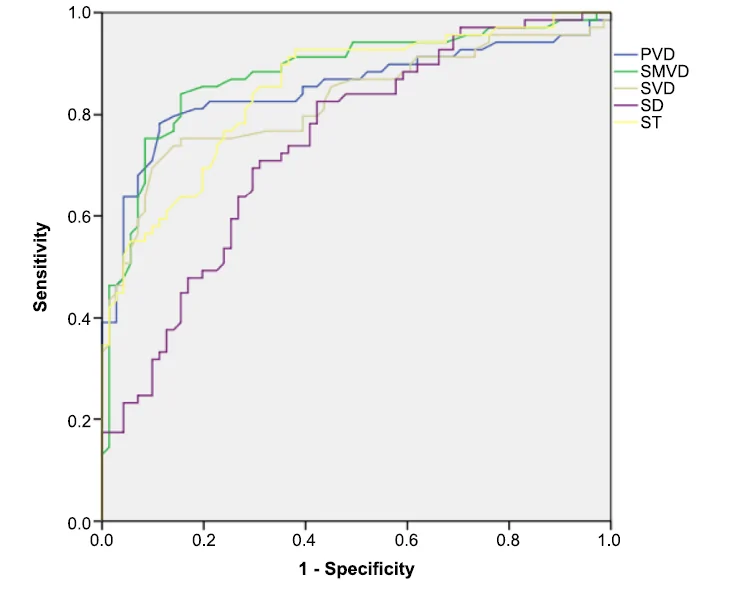
ROC curves for imaging parameters (PVD, SMVD, SVD, SD, and ST) in predicting PHGD. AUCs: PVD 0.852, SMVD 0.880, SVD 0.825, SD 0.742, ST 0.849.

**Table 1:** Clinical characteristics of the study population.

**Characteristics**	**PHGD group (n=69)**	**Non-PHGD group (n=71)**	***p*-value**
Age (years)	66.6 ± 10.4	69.3 ± 9.6	0.117
Sex (male/female)	34/35	36/35	0.866
**Cirrhosis etiology**			
Hepatitis B-related	27 (39.1%)	17 (23.9%)	0.052
Primary biliary cirrhosis	8 (11.6%)	3 (4.2%)	0.126
Alcoholic cirrhosis	7 (10.1%)	2 (2.8%)	0.093
Autoimmune cirrhosis	2 (2.9%)	0 (0%)	0.242
Schistosomiasis cirrhosis	1 (1.4%)	5 (7.0%)	0.208
Unknown etiology	24 (34.8%)	44 (62.0%)	0.001*

**Note:** *A *p*-value of <0.05 indicates statistical significance.

**Table 2:** Differences in laboratory test results between the PHGD group and the Non-PHGD group.

-	PLT(*10^*9^/L)	HB(g/L)	TP(g/L)	ALT(U/L)	AST(U/L)
PHGD	80.30±51.11	97.23±24.28	59.74±9.53	37.90±66.61	58.90±94.14
Non-PHGD	136.66±59.35	125.72±22.36	65.50±8.86	29.82±24.62	32.39±19.67
t-value	-6.026	-7.215	-3.702	0.947	2.291
*p*-value	0.000*	0.000*	0.000*	0.346	0.025*

**Abbreviaitons:** HB: Hemoglobin; PLT: Platelets; ALT: Alanine aminotransferase; AST: Aspartate aminotransferase; TP: Total protein; *p* < 0.05 indicates statistical significance.

**Table 3:** The diagnosis efficiency of laboratory test results and imaging findings.

-	Cut-off	AUC (95%CI)	Sensitivity (%)	Specificity (%)
PLT (*10^*9^/L)	97.50	0.797 (0.723-0.871)	77.5	71.0
HB (g/L)	113.50	0.805 (0.734-0.876)	76.1	71.0
TP (g/L)	63.63	0.685 (0.597-0.773)	63.4	72.5
AST (U/L)	29.5	0.642 (0.551-0.733)	59.4	57.7
PVD (mm)	14.35	0.852 (0.783-0.920)	81.2	81.7
SMVD (mm)	11.75	0.880 (0.821-0.940)	84.1	84.5
SVD (mm)	10.35	0.825 (0.754-0.897)	75.4	84.5
SD (mm)	123.85	0.742 (0.661-0.823)	71.0	69.0
ST (mm)	47.35	0.849 (0.786-0.913)	76.8	76.1

**Abbreviaitons:** HB: Hemoglobin; PLT: Platelets; ALT: Alanine aminotransferase; AST: Aspartate aminotransferase; TP: Total protein; PVD: Portal vein diameter; SMVD: Superior mesenteric vein diameter; SVD: Splenic vein diameter; SD: Splenic diameter; ST: Splenic thickness; AUC: Area under the Curve; CI: Confidence interval.

**Table 4:** Differences in imaging findings between the PHGD group and the non-PHGD group.

-	PHGD (n=69)	Non-PHGD (n=71)	T/χ2-value	*p*-value
PVD (mm)	16.66±3.62	12.70±1.90	8.097	0.000*
SMVD (mm)	13.92±2.87	10.33±1.82	8.814	0.000*
SVD (mm)	12.78±4.49	8.74±1.84	6.941	0.000*
SD (mm)	138.60±28.23	112.10±27.58	5.617	0.000*
ST (mm)	58.39±15.44	39.49±10.15	8.535	0.000*
Ascites	-	-	71.499	0.000*
Present	60	11	-	-
Absent	9	60	-	-
Varicose veins	-	-	66.381	0.000*
Present	65	19	-	-
Absent	4	52	-	-
Thrombosis	-	-	21.254	0.000*
Present	18	0	-	-
Absent	51	71	-	-

**Abbreviaitons:** PVD: Portal vein diameter; SMVD: Superior mesenteric vein diameter; SVD: Splenic vein diameter; SD: Splenic diameter; ST: Splenic thickness; *p* < 0.05 indicates statistical significance.

## Data Availability

The data supporting the results of this study will be available from the corresponding authors [J.W.] and [Z.W.] upon reasonable request.

## LIST OF ABBREVIATIONS

ALT: Alanine aminotransferase
AST: Aspartate aminotransferase
AUC: Area under the curve
CSPH: Clinically significant portal hypertension
CT: Computed tomography
HB: Hemoglobin
HVPG: Hepatic venous pressure gradient
MSCT: Multi-slice spiral computed tomography
NIEC: New Italian Endoscopic Club
PH: Portal hypertension
PHC: Portal hypertensive colopathy
PHG: Portal hypertensive gastropathy
PHGD: Portal hypertensive gastrointestinal disease
PHE: Portal hypertensive enteropathy
PLT: Platelet count
PVD: Portal vein diameter
ROC: Receiver operating characteristic
SD: Splenic diameter
SRT: Stool routine test
ST: Splenic thickness
SMVD: Superior mesenteric vein diameter
SVD: Splenic vein diameter
TNF-α: Tumor necrosis factor-alpha
TP: Total protein

## STANDARDS OF REPORTING

STROBE guidelines were followed.
